# MAGIC: once upon a time in consent management—a FHIR^®^ tale

**DOI:** 10.1186/s12967-018-1631-3

**Published:** 2018-09-14

**Authors:** Martin Bialke, Thomas Bahls, Lars Geidel, Henriette Rau, Arne Blumentritt, Sandra Pasewald, Robert Wolff, Jonas Steinmann, Tobias Bronsch, Björn Bergh, Galina Tremper, Martin Lablans, Frank Ückert, Stefan Lang, Tarik Idris, Wolfgang Hoffmann

**Affiliations:** 1 Institute for Community Medicine, Department Epidemiology of Health Care and Community Health, University Medicine Greifswald, Ellernholzstr. 1-2, 17475 Greifswald, Germany; 2 Independent Trusted Third Party, University Medicine Greifswald, Ellernholzstr. 1-2, 17475 Greifswald, Germany; 3 Technology, Methods and Infrastructure for Networked Medical Research (TMF), Charlottenstrasse 42/Dorotheenstrasse, 10117 Berlin, Germany; 4 Institute for Medical Informatics and Statistics, Kiel University and University Medical Center Schleswig–Holstein, Campus Kiel, Arnold-Heller-Straße 3, 24105 Kiel, Germany; 5 Department Medical Informatics for Translational Oncology, German Cancer Research Center (DKFZ), Im Neuenheimer Feld 280, 69120 Heidelberg, Germany; 6 Technical Committee FHIR, HL7 Deutschland e.V, Anna-Louisa-Karsch-Str. 2, 10178 Berlin, Germany; 7 InterComponentWare AG (ICW), Altrottstr. 31, 69190 Walldorf, Germany

**Keywords:** Medical data management, Informed consent, General data protection regulation, FHIR, IHE, APPC, Research network, Web application, gICS, Clinical trials, Patient rights, Hospital information systems

## Abstract

**Background:**

The use of medical data for research purposes requires an informed consent of the patient that is compliant with the EU General Data Protection Regulation. In the context of multi-centre research initiatives and a multitude of clinical and epidemiological studies scalable and automatable measures for digital consent management are required. Modular form, structure, and contents render a patient’s consent reusable for varying project settings in order to effectively manage and minimise organisational and technical efforts.

**Results:**

Within the DFG-funded project “MAGIC” (Grant Number HO 1937/5-1) the digital consent management service tool gICS was enhanced to comply with the recommendations published in the TMF data protection guideline for medical research. In addition, a structured exchange format for modular consent templates considering established standards and formats in the area of digital informed consent management was designed. Using the new FHIR standard and the HAPI FHIR library, the first version for an exchange format and necessary import-/export-functionalities were successfully implemented.

**Conclusions:**

The proposed exchange format is a “work in progress”. It represents a starting point for current discussions concerning digital consent management. It also attempts to improve interoperability between different approaches within the wider IHE-/HL7-/FHIR community. Independent of the exchange format, providing the possibility to export, modify and import templates for consents and withdrawals to be reused in similar clinical and epidemiological studies is an essential precondition for the sustainable operation of digital consent management.

## Background

### Informed consent in the times of GDPR

Medical research increasingly relies on processing patients’ identifying and medical data. When Plato wrote “*I shall assume that your silence gives consent*” [1], the legal permissibility of this form of “opt-out consent” was not an issue as it is nowadays—especially, in the context of healthcare and medical research.

The EU General Data Protection Regulation (GDPR) protects the rights of the data subject—in research: the concerned person—with regards to the right to privacy and related freedoms. Study participants and patients decide how and with whom their medical information is shared [2]. In this context, the informed consent is an important utility for researchers to ensure compliance with legal data protection obligations.

If the purpose of the data processing is not specifically determined by a legal basis (GDPR Art. 6 (3)), data collection for research purposes requires an informed consent (GDPR Art. 6 (1) lit. a) [3]. An implicitly given consent of the participant following the opt-out principle is legally not permissible. Recital 32 (Conditions for consent) indicates that *“neither silence, pre*-*ticked boxes nor inactivity of a participant constitutes consent”* [4]. According to Recital 32 of the GDPR consent requires to be given “*freely, specific, informed and unambiguous*” [4] (opt-in principle) and can be given by *“electronic means”* [4]. Moreover, the consent regarding data processing for different purposes (e.g. processing of research data, or processing of biological samples), “*needs to be given separately for each data processing activity“*([5], p. 10) in order to guarantee the participant’s rights and necessary freedom. Furthermore, withdrawing the informed consent should be as easy as giving it (GDPR Art. 7 [6]).

These requirements strongly suggest a modular informed consent conforming to the consent model “opt-in with restrictions” ([7], p. 7). The data controller needs to set up efficient yet transparent organisational mechanisms to implement the participant’s rights and freedoms (GDPR Art. 5 (1)). Thus, informed consents help the data controller to fulfill their obligation to provide proof of participant’s consent to supervisory authorities, pursuant to GDPR Art. 7 (1) [6].

### Research upon clinical care data requires a multitude of informed consents

Research based on medical data comprises the aggregation of various datasets coming from most diverse data sources and originating from different episodes of the treatment process as well as from different periods of a person’s life. To permit finding answers to a wide range of research questions, the applied consent has to reflect the heterogeneous characteristics of these datasets in terms of time and content.

The University Medicine Greifswald (UMG) replaces and enhances its Hospital information system (HIS; German: KAS [8]), to a novel integrated IT infrastructure: the KAS+. It enables access to clinical care data for health services research and to support clinical and epidemiological studies. It is legally permissible to use the patient’s data for research purposes based on a given consent. Considering more than 35,000 inpatient cases of this hospital per year, a traditional paper-based documentation and management of the patient’s informed consent conflicts with the goal to use collected data for a large number and variety of research projects. This requires process automation. The significant number of clinical studies running in parallel in most academic hospitals (typically 50–220 clinical studies per hospital) calls for workflow-integrated, digital solutions of consent management. Pertinent regulations and data quality requirements also foster these developments.

Research initiatives like the German National Cohort (NAKO) [9], the German Cancer Consortium (DKTK) [10], consortia of the Medical Informatics Initiative (MI-I) and EU-funded research projects such as the Baltic Fracture Competence Centre (BFCC) [11] depend on scalable, digitalized and automated solutions. Consent processing has to be integrated with process workflows of inpatient and outpatient admission of the patient (system integration). Gathering and documenting the patients will have to be time-efficient and, thus, consistently digital. Processing of informed consents in systems, which technically as well as organisationally separate person identifying data from clinical data, is required to comply with data protection regulations [12]. This precondition requires separate infrastructures and well-defined interfaces. The responsible data controller needs a way to easily determine (i.e. digitally query) the current status of a patient’s consent regarding a specific aspect (e.g. usage for a specific research purpose or contact policy). Such requirements are satisfied in current projects using centralised facilities such as a Trusted Third Party (TTP [13]) that provides algorithms and online interfaces to support a project’s data management with the needed services. At the same time, this centralised facility allows for immediate application of withdrawals as well as systematic monitoring and timely quality checks on received consents.

Consequently, the integration of a digital consent management solution in a multitude of health service research projects—in-house, third-party funded or co-operation projects—relies on standards developed for interfaces (e.g. Integrating the Healthcare Enterprise (IHE), Fast Healthcare Interoperable Resources (FHIR)).

### Definition, documentation, and application of informed consents

The formulation of participant information and informed consent documents for the study requires the description of the study’s individual setting as well as planned data processing procedures. These descriptions have to be both, legally reliable and generally understandable. The Technology, Methods, and Infrastructure for Networked Medical Research e.V. (TMF), an umbrella organisation for networked medical research in Germany, provides assistance regarding the content and structure of formulating participant information and informed consent documents. The TMF’s “*wizard to prepare patient information and consent*” (PEW [14]) follows a wiki-based approach and was essentially revised in 2017 [15] with regards to technical aspects. The PEW uses numerous verified text elements linked to the respective legal requirements to reduce the author’s efforts.

Modular informed consents can be managed using the generic Informed Consent Service (gICS), developed by the Institute for Community Medicine of the University Medicine Greifswald (UMG) [16]. By design, gICS fosters a centralised electronic consent management ([7], p. 8) and supports the “*opt*-*in with restrictions*” consent model ([7], p. 7) while providing support for both paper-based and purely digital consent workflows. However, using gICS for consent management does not guarantee full data protection compliance. For example, gICS does not support drafting a study consent to address all data protection rights of a patient and does not facilitate the ethical approval of a study consent by a responsible ethics committee. At the time of writing, the consent management tool is used in eight research projects, including the NAKO, the German Centre for Cardiovascular Research (DZHK) [17], the BFCC and the Greifswald Approach to Individualized Medicine (GANI_MED) project [18]. These projects use gICS as an essential component of a Trusted Third Party (TTP) [13] as part of a complex research infrastructure. Using gICS, approximately 203,000 informed consents and more than 640 withdrawals (cumulative values, last updated January 2018) are documented, using a structured and automatically processible approach. The open source tool gICS is licensed under GNU Affero General Public License Version 3 (AGPLv3), free of charge and downloadable via the homepage of the MOSAIC project [19], Github [20] and the official docker-repository of the TMF [21].

Consider the three central use cases of consent management (cf. Table 1): a suitable exchange format to easily adopt the PEW’s *(A) Consent Definitions* as a consent template into gICS was missing so far. Based on consent templates, gICS supports to subsequently start the *(B) Digital Documentation and Tracking* of informed consents and withdrawals. *(C) Application of the informed consent* on the participant’s data can be automated more fully by using an access control language to describe a consent’s implications as standardized in IHE Advanced Patient Privacy Consent (APPC) [22] for a limited domain. Consent application is a continuous process requiring an up-to-date and transparent documentation of consents and withdrawals. Consent application was not part of the functional range of gICS. At the moment, applying the consent is a separate process mostly handled by the systems holding the relevant (medical) data.

**Table 1:** The three central use cases of consent management

No.	Consent use case	Description
A	Definition	Consent definition describes the consent with regards to content, form, and layout (e.g. content modules, descriptive and legal texts, the order of modules, necessary answer options). It can be understood as a template for specific consent document instances
B	Documentation and tracking	Consent documentation and tracking imply a digital system that documents and tracks the agreement of a specific patient to a clearly defined consent and/or withdrawal, i.e. who has consented when with regards to what and/or which part of the consent was if so, withdrawn
C	Application	Consent Application and enforcement requires an additional technical understanding of the consent definitions. Its goal is to automatically apply a given consent or withdrawal (and respective rules), so that all relevant systems reflect the patient’s wishes, ideally without further manual intervention

Having in mind the current dissemination of gICS, the growing complexity of GDPR-compliant consents and the increasing number of project sites as part of the Medical Informatics Initiative, the homogeneous composition of these three (cf. Table 1) currently detached consent management aspects (Definition, Documentation and Tracking and Application) becomes an important objective in medical research. Aligning them more closely promises significant potential in process automation. This could reduce efforts for definition, documentation, and application of an informed consent in current and future research networks.

### The MAGIC project

The TMF data protection guideline enables medical research projects to establish necessary research infrastructures in compliance with data protection regulations [12]. The guideline defines common requirements in the fields of identity management, administrations of rights, consent management (cf. TMF data protection guideline, sections 6.1, 6.2 and 6.6.6 [12]) and authentication/authorisation. In the context of the DFG-funded project “MAGIC”, selected software solutions are enhanced in order to fulfill these requirements.

## Objectives

**Fig. 1:**
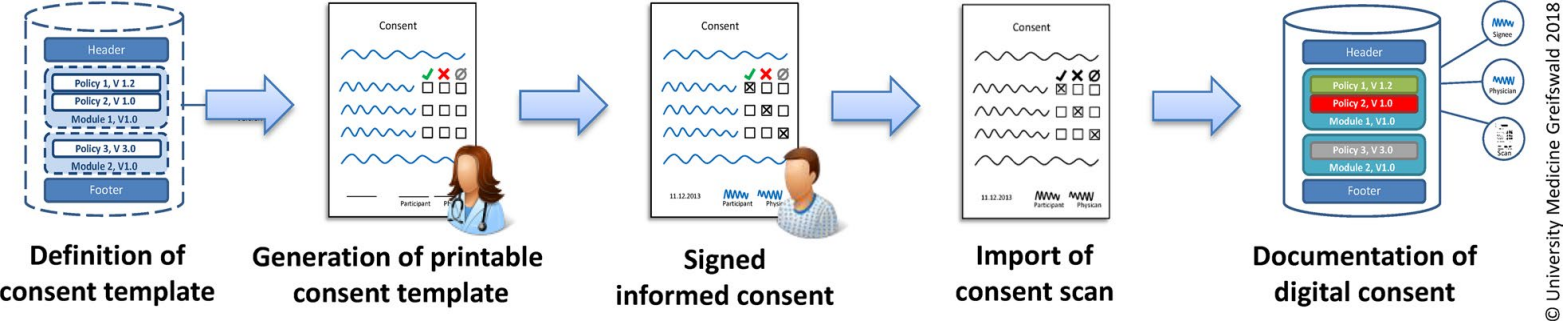
A structured exchange format for informed consent templates fosters the convergence of paper-based and digital informed consent management with gICS

The aim of the MAGIC-partner Institute for Community Medicine of the UMG is to enable research projects with up-to-now purely paper-based consent procedures to use gICS and, thus, to benefit from the advantages of digital consent management. For this purpose, gICS will be enhanced to import and export existing consent templates. The goal is to improve support for *Consent Definition* and *Consent Documentation*. This includes the generation of printable consent templates and support for an automatic parsing of paper-based consent scans (cf. Fig. 1). Motivated by a multitude of studies in the context of the KAS+ project, document templates for consents and withdrawals will be reusable for varying project settings. It will also be possible to include consent definitions originating from the PEW into gICS with minimal efforts.

To facilitate these goals, this article describes the design and implementation of a structured exchange format for modular consent templates considering established standards and formats in digital informed consent management.

## Methods

### Assessing the benefit of a modular consent structure

Participants of a study have to be able to decide on their own which aspect of a research study (as described in an informed consent) is acceptable and which aspect they would like to decline. The alternative approach, where participants that disagree with any aspect of a study are fully withdrawn, can quickly impair the outcome of a research project or study. Thus, consents, as well as withdrawals, should be modular by design.

Experiences gathered within the trusted third party of the NAKO show a positive impact on the withdrawal behaviour of participants. If the participant is able to only withdraw selected elements of a consent (partial withdrawal, e.g. to re-contact the concerned person), the participants tend to refrain from a full withdrawal [23]. Within the NAKO 544 withdrawals have been documented until January 2018, including a ratio of 69.8% (N = 380) for partial withdrawals. In detail, 25.2% (N = 137) of all documented withdrawals refer to the consent module “re-contact” only. Due to providing the possibility of partial withdrawals the participant’s demand “not to bother her/him in the future” will be satisfied, while already collected research data is still available for research (as consented beforehand). Consequently, studies and their participants benefit from modular consents.

**Fig. 2:**
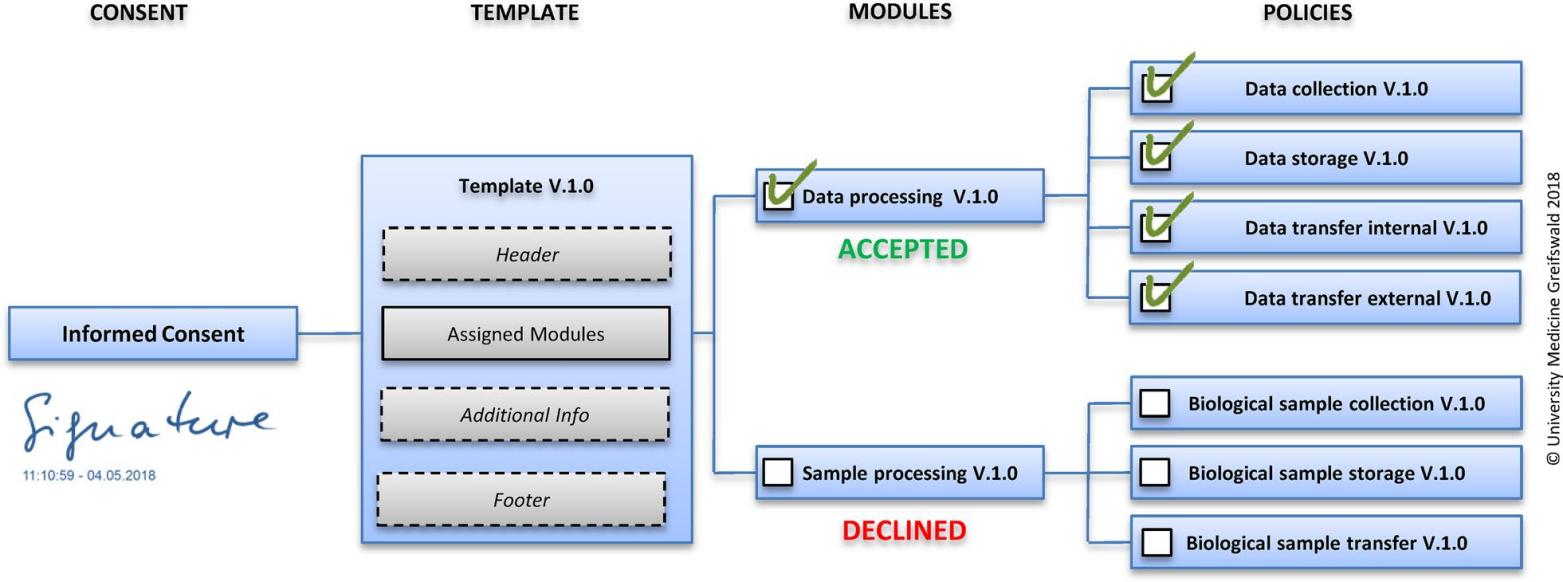
Relationship between policy, module, template and informed consent (based on [16])

Modular consents are based on policies (cf. Fig. 2). A policy represents a to-be-consented fact, e.g. a single step of data processing. In practice, aggregating related or logically coherent policies into modules has proven to be successful. For example, policies for capturing, transferring and storing data might be combined in a module “data processing”. Each module is attributed with formatted text such as the relevant part of the informed consent document. Modules enable a participant to consent to a multitude of related policies at the same time.

Within gICS, each documented informed consent is based on a versioned template. This template describes the structure of the consent form and contains assigned modules. Each module refers to an individually assigned set of policies. Changing the acceptance status of a module (accepted, declined, withdrawn, etc.) directly affects related policies. The template also contains complementary information for the consent document (inter alia, the order of modules, the definition of obligatory modules, header, footer and free text fields).

Policies, modules, and templates are managed in contextual domains. For reasons of simplicity, domains may be projects or clients and provide context-related information such as project-logo and details of versioning. All this is required to satisfactorily depict a consent digitally. Each consent documents the participant’s wish based on a versioned template. Depending on the configuration, this digital informed consent can include not only the signatures of the participant and the physician but also a scan of the paper-based consent document (see Fig. 2).

### Standardising a modular informed consent

#### Step 1: Identifying relevant information

**Fig. 3:**
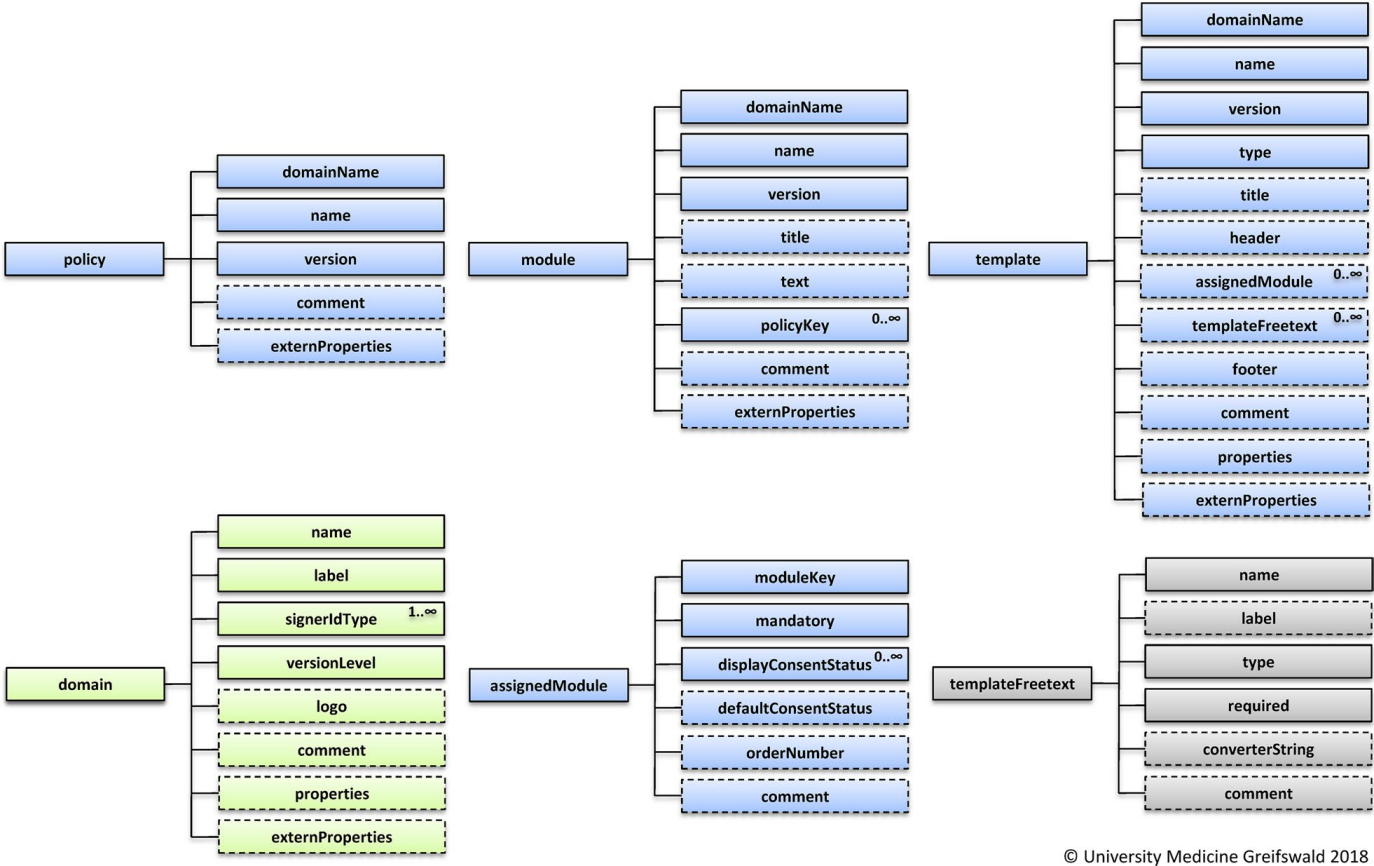
Overview of relevant information to ex- and/or import all consent templates for a study or research project (optional elements with dashed outlines): green elements are used to depict project information, while blue elements describe core elements. Grey elements relate to additional information

Based on published requirements regarding informed consents [24], existing gICS source code and new requirements in current application projects (NAKO, DZHK, BFCC) necessary information for the automatic creation of domains, policies, modules, and templates were compiled to identify relevant information for the new exchange format. A summary of this information is depicted in Fig. 3.

#### Step 2: Orienting on common standards and formats

To reduce efforts regarding implementation and to achieve a preferably broad utilisation of the exchange format, established standards and formats should be taken into account. To be able to assess the suitability of these common standards and techniques, necessary requirements were compiled (cf. Table 2).

**Table 2:** Requirements for a consent template exchange format

Requirement	Description
Portability/extensibility	Essential gICS-information have to be transformable into the selected standard/format, or the selected standard/format offers necessary degrees of extensibility
No license fees	gICS is published under open source license (AGPLv3) and, thus, is free of cost for users. As a consequence, implementation and use of the proposed exchange format must not raise additional costs in terms of license fees for developers as well as users
Comprehensive developer support	To minimise implementation efforts, the selected standard/format has to offer a comprehensive support for developers and related development procedures including contemporary (web-based) technologies, support for parsing, validating, encoding and decoding the proposed format. Moreover, the use of maven repositories and an active developer community (e.g. blogs, chats or direct contact partners) for further inquiries or bug reports are preferable
Dissemination	The selected format/standard has to be already implemented in the scientific community (i.e. not being in conceptual, draft or prototypic state) in order to allow an implementation during the runtime of the MAGIC project

With regards to these requirements several common standards, profiles and formats were assessed—a short summary is given in Table 3.

**Table 3:** Summary of evaluated common standards, profiles, and formats

Name	Description
HL7 FHIR (standard)	Health Level Seven International (HL7) Fast Healthcare Interoperable Resources (FHIR), a REST-based standard for interoperability in healthcare to access distributed information (e.g. patient, medication and treatment) in a uniform, open format using JSON and XML [30]
HL7 v2 (standard)	A standard for information transfer and to support system integration processes, e.g. to exchange patient-, performance- or finding-related information within hospitals [45]
HL7 CDA (standard)	Clinical Document Architecture (CDA) is a standard for document-based information exchange in primarily clinical use cases. CDA offers the possibility to combine human-readable and machine-readable contents [46]
IHE BPPC (profile)	The IHE profile Basic Patient Privacy Consents (BPPC) allows basic and non-recurring documentation of a patient’s consent regarding the exchange of his/her data between cooperating facilities (e.g. hospitals). [27]
IHE APPC (profile)	The IHE profile Advanced Patient Privacy Consents (APPC) is a profile [28] describing how to use a domain specific language for access control rules to create a policy document. It focusses on how to reference data communicated using other IHE profiles (e.g. IHE XDS) in that language. It also contains rules for how to transmit such a policy document using IHE document sharing profiles. While the underlying standards for these highly structured policy documents enable automatic enforcement, the profile itself does not contain any of those transactions, only the policy document structure, and metadata [47]
XML (format)	Extensible Markup Language (XML), a format for the structured description of data [29]
JSON (format)	JavaScript Object Notation (JSON), a simple format for data. No additional functionality [29]

##### HL7 v2 and HL7 CDA

Not all Health Level Seven International (HL7) standards are freely available. If HL7 v2 or HL7 CDA (HL7 Clinical Document Architecture) had been used to map necessary gICS-information (cf. Fig. 3), license fees had to be paid [25, 26]. Developers and users of a software, which applies the HL7-standard in order to read and modify CDA-documents, have to be “organisational members” of the HL7-consortium. Thus, the integration of these HL7 standards into the free software solution gICS for consent management might have a negative impact on its future usability within the scientific community.

##### IHE BPPC and APPC

Large health infrastructures, such as “longitudinal patient record systems” or “Health Information Exchanges”, use the IHE-profile “Basic Patient Privacy Consents” (BPPC) to document the patient’s consent to allow data transfer between connected systems [27]. Based on the BPPC concepts, the follow-up profile “Advanced Patient Privacy Consents” (APPC) is currently being field tested. APPC focusses on access control rules resulting from consents [﻿28﻿]. Therefore, APPC provides the required mechanisms to support automated enforcement of specific policies regarding the managed research data, according to the patient’s consent. For example, a patient’s policy document could grant access to research data originating from a specific hospital only [22]. In contrast to BPPC, APPC is more precise with regards to granularity and provides more flexibility. However, both profiles do not focus on *Consent Definition* and do not contain templating mechanisms or capabilities to link legal text, user-friendly text, and access control rules.

##### XML and JSON

The Extensible Markup Language (XML) and the JavaScript Object Notation (JSON) are popular and lightweight formats for data transfer and easy to adopt concerning individual project requirements [﻿29﻿]. However, both formats require significant additional efforts to specify structure and form of contained information in terms of profiles.

##### HL7 FHIR

The new HL7 standard FHIR is a (free) part of the HL7 family of standards and receives great appreciation and dissemination in the healthcare sector worldwide and, especially, in the EU and Germany. FHIR leverages common web technologies to offer access to distributed data in healthcare infrastructures [30] and combines quality characteristics of HL7 v2 and CDA [31]. Thus, FHIR might generally be used for *consent documentation and tracking*. The resource FHIR Consent [32] intends to depict the contractual character of consents between patient and treating facility (hospital). It allows overall documentation of consent in terms of a defined set of permissible actions, which are based on selected data processing procedures (collect, access, use, disclose, correct). Depicting a partial consent using an opt-in/opt-out approach with an optional description of exceptions is in principle supported but limited regarding formalisation. Currently, the resource FHIR Consent is in an early stage of development (“*Mature 1*” [33]). It does not handle complex consent scenarios, e.g. the NAKO with about 110 different consent modules, well. Also, the current form of FHIR Consent does not fully facilitate *Consent Definition* (in terms of consent templates). Due to the limited granularity of FHIR Consents (regarding permissible actions and applicable rules), an automated conversion into IHE APPC for enforcement is currently hard to achieve. Nevertheless, this may be achievable in the future, since both, FHIR Consent as well as IHE APPC, refer to the Extensible Access Control Markup Language (XACML) to describe technically enforceable rules for access control.

After evaluating the common profiles, formats and standards, the *Consent Definition*, the *Consent Documentation and Tracking* as well as the *Consent Application* currently cannot be aligned.

#### Step 3: Developing an exchange format with HAPI FHIR

FHIR is a fast growing standard for the exchange of healthcare related data both in medical care and research. The application of FHIR is not limited to specific scenarios or use cases. Thus, current research initiatives show great interest to evaluate the emerging standard and to share practical experiences [34]. The new FHIR Standard is currently a “Standard For Trial Use” (STU3, April 2017). It will become a normative standard shortly (FHIR R4, planned for December 2018).

Existing FHIR resource definitions are provided for free and are almost arbitrarily extensible (via FHIR extensions). Definition as well as provision of new requirements and necessary modifications of FHIR profiles can easily be accomplished using the available FHIR registries (e.g. Simplifier [35]) and profile editors (e.g. Forge [36]). New FHIR resources can be proposed and, after testing by the community and proving their value, they can be integrated into the FHIR standard. The implementation of FHIR profiles is a task to be performed by developers of the respective research projects. This task is considerably simplified by existing free developer tools like HAPI FHIR (HL7 application programming interface for FHIR) [37].

The new FHIR standard is conforming to the requirements listed in Table 2. A first version of the exchange format, addressing relevant information to share well-structured consent templates (cf. Fig.  3), was successfully implemented using the HAPI FHIR. Supplied validators and converters, as well as the active developer community (in terms of blogs, chats, and forums), helped to minimise the necessary implementation effort for first feasibility tests.

## Results

**Fig. 4:**
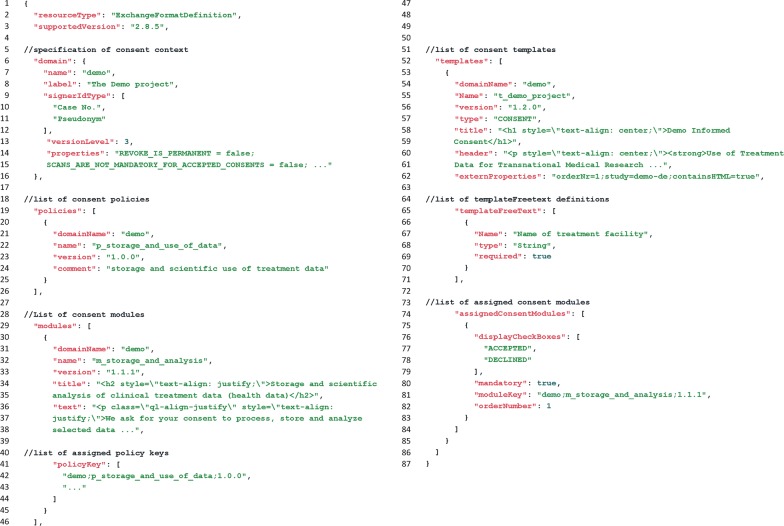
Simplified JSON-representation of the exchange format (exemplary with a reduced set of policies and modules)

This work included the compilation of necessary information to depict a consent template (cf. Fig. 3) and the evaluation of common standards, formats and popular profiles (cf. Table 3) regarding necessary requirements (cf. Table 2). The proposed structured exchange format was implemented by the Institute for Community Medicine of the University Medicine Greifswald using the free HAPI FHIR [37] for first feasibility tests. It is oriented towards the technical guidelines of the new FHIR standard [30] and consists of six new datatypes. To encapsulate these contents for a simplified data transfer, a new resource “ExchangeFormatDefinition” was defined. For illustration Fig. 4 shows a simplified representation (with a reduced number of policies, modules, etc.) of the exchange format.

New functionalities were implemented in gICS to support import and export of this FHIR-based exchange format, which can be accessed directly using the provided web-service or the web-based user interface. Calling the import mechanism invokes validation of the specified exchange format. Configurations for domains, policies, modules, and templates included in this exchange format will be instantly created within gICS or updated, if permissible.

The latest version of gICS (version 2.8.5) comprises functionalities to generate printable consent templates. As illustrated in Fig. 1, gICS was enabled to process consent scans directly and to automatically detect the consent template used for the informed consent. Thus, the intended support for paper-based consent procedures could be improved with the help of gICS within the MAGIC project (see Fig. 1).

One of the challenges of the KAS+ project mentioned earlier in this paper is to provide mechanisms supporting the significant number of 50–220 parallel small- and mid-sized clinical studies in typical academic hospitals. Recording of a patient’s consent, as well as automated queries of the current status of specific consent modules during data management and data usage, are key elements in such fully integrated and automatised setups. A simple free-text programming of monolithic text blocks or digitalisation by archival of unstructured PDF scans of paper-based consents are no valid options for digital consent management. Instead, the right digitalisation strategy is to build consents from a template database and adapt only the specifics of a study, then simply import the result into the existing consent management tools. It addresses the operational needs when a variety of studies is running in parallel. This way, comparability of the contents of the various study consents is achieved, data quality is improved, and compliance with regulatory requirements such as the GDPR can be assured and audited.

## Discussion

Already in 2015, during the evaluation of the applied European Regional Development Funds (ERDF) by the state of Mecklenburg-Western Pomerania, the University Medicine Greifswald successfully demonstrated using gICS for the integrated documentation, processing, and application of patients’ consents. The current implementation of the KAS+ infrastructure benefits from the fully digital process chain. As a result, the KAS+ research platform [8] continuously compares inclusion criteria of studies with properly consented research data. If a patient matches the inclusion criteria of a specific study, the research platform validates the consent status of the respective patient and checks the consent module for re-contact. Following the principles of Trinczek et al. [38], the research platform then automatically generates and adds a recruitment proposal on the worklist for the staff of the respective clinical study within the primary clinical system. The responsible study nurse, for instance, just needs to confirm the proposal to start the digital documentation of the patient’s consent and include the patient in another study.

The developed exchange format was presented at the annual congress of the German Association for Medical Informatics, Biometry and Epidemiology e.V. (GMDS) in 2017 [39]. In addition, a possible integration of the exchange format into the latest version of the TMF’s PEW [15] has been actively suggested by the developers of gICS but requires further consolidation with the responsible developers of the TMF’s PEW.

As a result of the short presentation at the GMDS, the first version of this exchange format was introduced to the community at the periodically arranged “Forum for Interoperability of HL7 Germany, IHE Germany, German Association of Health IT Vendors (bvitg e.V.) and German Institute for Standardisation (DIN) (Forum for Interoperability)” [40]. Aims of the forum are to discuss and consolidate present approaches to ensure interoperability for a range of applications within the healthcare sector. An important part of the discussion with representatives of HL7 FHIR was the practical concept and benefit of using modular informed consents in cohort studies and registries. As a first achievement, a central communication platform for consent management in German-speaking countries (Germany, Austria, Switzerland) is now available as part of the official FHIR chat [41]. Developers and interested parties are welcome to participate. In a second meeting of the Forum for Interoperability, representatives of HL7, IHE, FHIR, selected MI-I consortia (MIRACUM [42], SMITH [43], HiGHmed [44]) and industry showed intent to harmonise existing standards and profiles (HL7, FHIR, IHE APPC) with regards to consent management.

The consensus was reached on the existence of three essential, but currently separated, use cases of consent management: *the Consent Definition, the Consent Documentation, and Tracking* as well as *the Consent Application* (cf. Table 1).

The presently separated consideration of these three aspects in existing tools (e.g. PEW, gICS) and profiles (e.g. IHE APPC, HL7 FHIR Consent) should be resolved. This could be achieved by appropriate new or modified profiles and standards. The consolidation process has to take into account the described modular approach for informed consents [16] and should make use of technologies already common to the considered profiles, such as XACML.

## Conclusions

A prerequisite for printing an “empty” consent (a consent template to gather a patient’s informed consent) is the definition of a consent template in form of structure, content, and layout (cf. Figure 1). To reduce necessary efforts (from a gICS perspective), (1) reuse of consent templates in similar project settings as well as (2) linking gICS and the TMF’s PEW [14] should be facilitated by the MAGIC project. A goal of the MAGIC project at the University Medicine Greifswald was to design and implement a structured exchange format for modular consent templates. The format had to take common standards in the area of digital informed consent management into account.

The developed exchange format for consent templates represents a “work in progress”. As of today, the current implementation is not part of the official FHIR standard. Nevertheless, it is a starting point for discussions and endeavours in informed consent management. First consolidations as part of the Forum for Interoperability [40] were already successful. An essential result is the establishment of a new working group “Consent Management” in March 2018 with representatives of FHIR, IHE, selected MI-I consortia, the industry as well as developers of the gICS.

Goals of the working group will be the consolidation and extension of existing standards and profiles (e.g. FHIR Consent, IHE APPC) based on the proposed modular concept of informed consents [24], the relevant information of the exchange format as described in Fig. 3, and the precise separation of consent template and informed consent. Thus, the technical harmonisation of HL7 FHIR (to document informed consents) and IHE APPC (for application and enforcement of the informed consent based on respective rules) shall be ensured in the long term. Results of the working group presumably will affect existing specifications and profiles of HL7 FHIR and IHE APPC or result in a new FHIR resource type and/or IHE profile with respect to the three use cases of consent management (as described above). An updated version of the exchange format, with regards to latest developments and the results of the working group, will be released afterwards.

With current endeavours in mind, the present implementation of the exchange format presumably has to be modified and/or extended with regards to future profiles. Sooner or later, managed informed consents and withdrawals within gICS shall be provided for simplified access using an FHIR-based REST-interface. Moreover, mapping of applicable consent policies and respective IHE APPC access control rules should be offered. The practical implementation of both aspects has to be aligned to “real-world” needs of the scientific community and has to be coordinated in close collaboration with representatives of the working group “Consent Management”.

In summary, using existing tools to efficiently implement the management of informed consents and withdrawals, whether paper-based or purely digital, in research projects and healthcare can be accomplished. From the first draft of the consent document to the automated enforcement of the patient’s consent on data management side, necessary procedures could be holistically improved by existing tools, standards and profiles in the area of consent management. Researchers can use a comprehensive consent management to remain in compliance with the legal requirements of the GDPR.

## Abbreviations

AGPL: GNU Affero General Public License
APPC: Advanced Patient Privacy Consent
BFCC: Baltic Fracture Competence Centre
BPPC: Basic Patient Privacy Consents
Bvitg: German Association of Health IT Vendors (Bundesverband Gesundheits-IT—bvitg e. V.)
CDA: Clinical Document Architecture
DFG: German Research Foundation (Deutsche Forschungsgemeinschaft)
DIN: German Institute for Standardisation (Deutsches Institut für Normung e.V.)
DKTK: German Cancer Consortium (Deutsches Konsortium für Translationale Krebsforschung)
DZHK: German Centre for Cardiovascular Research (Deutsches Zentrum für Herz-Kreislauf-Forschung e.V.)
FHIR: Fast Healthcare Interoperable Resources
GANI_MED: Greifswald Approach to Individualized Medicine
GDPR: EU General Data Protection Regulation
gICS: generic Informed Consent Service
GMDS: German Association for Medical Informatics, Biometry and Epidemiology e.V. (Deutsche Gesellschaft für Medizinische Informatik, Biometrie und Epidemiologie)
HiGHmed: Heidelberg-Göttingen-Hannover Medical Informatics (MI-I Consortium)
HIS: Hospital Information System
HL7: Health Level Seven International
IHE: Integrating the Healthcare Enterprise
JSON: JavaScript Object Notation
KAS+: Hospital information system with native research support (klinisches Arbeitsplatzsystem)
MI-I: Medical Informatics Initiative
MIRACUM: Medical Informatics in Research and Care in University Medicine (MI-I Consortium)
NAKO: German National Cohort
PEW: Patient information and consent—Wiki and Wizard
SMITH: Smart Medical Information Technology for Healthcare (MI-I Consortium)
TMF: Technology, Methods, and Infrastructure for Networked Medical Research e.V. (Technologie- und Methodenplattform für die vernetzte medizinische Forschung e.V.)
TTP: Trusted Third Party
UMG: University Medicine Greifswald
XACML: Extensible Access Control Markup Language
XML: Extensible Markup Language
